# First-Principles Study of Electronic, Optical Adsorption, and Photocatalytic Water-Splitting Properties of a Strain-Tuned BTe/PtS_2_ vdW Heterstructure

**DOI:** 10.3390/molecules31122057

**Published:** 2026-06-12

**Authors:** Wenming Cheng, Hao Pan, Yuxing Zhang, Jiaming Ni

**Affiliations:** 1 School of Automotive and Transportation, Shenzhen Polytechnic University, Shenzhen 518055, China; chengwenming@szpu.edu.cn; 2 School of Aircraft Engineering, Nanchang Hangkong University, Nanchang 330063, China; z3103785740@gmail.com; 3 School of Materials Science and Engineering, Nanchang Hangkong University, Nanchang 330063, China; nijiaming@nchu.edu.cn

**Keywords:** heterojunction, BTe/PtS_2_, bandgap, biaxial strain, optical properties, hydrolysis photocatalyst

## Abstract

The structural, electronic, optical, transport and photocatalytic properties of the BTe/PtS_2_ vdW heterojunction are investigated by the density functional theory approach. The results reveal that applying the tensile effect can significantly impact the material’s properties. The bandgap values of BTe, PtS_2_ and BTe/PtS_2_ vdW heterojunction are 1.599 eV, 1.756 eV and 1.19 eV, respectively. The bandgap decreases as the percentage of applied tensile effect increases. Moreover, the photocatalytic water decomposition of BTe/PtS_2_ vdW heterojunction is significantly broadened in the pH range compared with that of the two monolayers. In conclusion, the BTe/PtS_2_ vdW heterojunction can be used as an efficient photocatalytic material for optoelectronic devices and photocatalysis.

## 1. Introduction

The extensive use of traditional fuels has led to increasingly serious environmental problems, and the development of non-polluting renewable energy is an urgent task. Photocatalytic water decomposition (PWD) technology, which uses light energy to decompose water into oxygen and hydrogen, is an effective way to improve environmental pollution and solve the problem of energy shortage. In recent years, with the continuous upgrading of experimental techniques, more and more photocatalytic materials have been successfully prepared by researchers and have played a significant role in the photocatalysis field. Common photocatalytic materials include TiO_2_ [1], ZnO [2], CeO_2_ [3], BTe [4], PtS_2_ [5], etc. Among them, BTe (boron tellurium compound) and PtS_2_ (platinum disulfide), as new two-dimensional materials, have shown great potential in the fields of energy, catalysis, and electronics due to their unique electronic structures and excellent photoelectronic properties. The bbandgapof BTe is about 1.5 eV, which can effectively absorb visible light and is suitable for photocatalytic reaction. BTe has a high electron mobility, which is conducive to the separation and transport of photogenerated electron–hole pairs, and shows good chemical stability in photocatalytic reactions [6,7,8,9,10]. The band gap of PtS_2_ is about 2.0–2.5 eV, which is suitable for absorbing ultraviolet light and part of visible light, and it has abundant active sites on the surface, which can effectively promote the decomposition of water. PtS_2_ has excellent conductivity, which contributes to the rapid transport of photogenerated electrons [11,12,13,14]. By combining the advantages of the two, the BTe/PtS_2_ composites are able to achieve synergistic enhancement of performance, which has a wide range of applications, especially in the fields of photocatalysis, energy storage, and sensors [15,16,17,18]. Group-10 transition metal dichalcogenides (TMDCs) represent an intriguing subclass that has garnered extensive theoretical and experimental attention [19,20,21,22,23,24]. For instance, Ren et al. [25] demonstrated that combining PtS_2_ with Are creates a heterojunction with an impressive solar-to-hydrogen (STH) efficiency of 49.32%, marking a substantial improvement over the isolated components. Similarly, Ren et al.’s [26] investigations on the MoTe_2_/PtS_2_ vdW heterostructure revealed that specific stacking configurations can yield both type-I and type-II band alignments, establishing appropriate band edge positions for water-splitting and optoelectronic applications. Furthermore, Li et al. [27] reported that the InN/BTe heterojunction can drive the hydrogen evolution reaction (HER) spontaneously relative to the standard hydrogen electrode, achieving an outstanding optimal STH conversion efficiency of 17.92%.

The present work comprehensively explores the structural configurations, thermodynamic stability, and optoelectronic characteristics of the BTe/PtS_2_ van der Waals (vdW) heterostructure via DFT-based first-principles calculations. Evaluations based on cohesive energy and phonon dispersion confirm the robust energetic and dynamic stability of this composite. Furthermore, electronic band assessments reveal that the constructed heterostructure retains a semiconducting nature, possessing appropriate bandgap dimensions and ideal band alignments, thereby validating its viability as a highly efficient photocatalyst for water splitting.

## 2. Computational Methods

All first-principles calculations in this study were executed within the framework of density functional theory (DFT), utilizing the CASTEP software (version Materials Studio 2020) package [28]. To accurately depict the interactions between core ions and valence electrons, the projector augmented-wave (PAW) method was adopted [29]. Meanwhile, the exchange-correlation functional was parameterized using the generalized gradient approximation (GGA) formulated by Perdew Burke Ernzerhof (PBE) [30]. We established a kinetic energy cutoff of 500 eV for the plane-wave basis [31]. Brillouin zone integration was performed using a well-distributed 9 × 9 × 1 Monkhorst-Pack k-point mesh [32]. The convergence thresholds for geometry optimization were rigorously set at 10^−5^ eV for total energy and 10^−2^ eV/Å for maximum residual forces on each atom [33]. Additionally, to eliminate artificial boundary interactions between adjacent layers, a minimum vacuum space of 20 Å was inserted along the perpendicular Z-direction.

## 3. Results and Discussion

**Figure 1:**
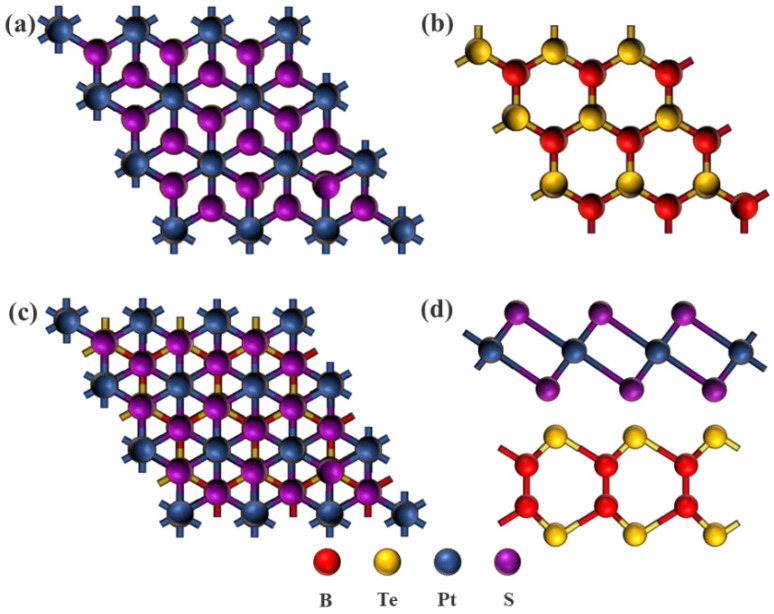
Configuration of (**a**) PtS_2_ monolayer, (**b**) BTe monolayer and (**c**) the top view of PtS_2_/BTe monolayer vdW heterojunction. (**d**) The side of PtS_2_/BTe vdW heterojunction.

The top and side views of the BTe and PtS_2_ monolayers are shown in Figure 1, from which it can be seen that both of them are composed of a six-membered ring honeycomb structure. As shown in Figure 1a, the BTe monolayer is a four-atom layer consisting of two atoms, B and Te, which can be regarded as a ‘sandwich’ structure consisting of B-Te, B-B, and B-Te layers stacked sequentially. As shown in Figure 1b, the Pt atoms in the PtS_2_ monolayer are covalently bonded to the two neighboring S atoms. The calculated parameters are listed in Table 1. The lattice constants of the BTe and PtS_2_ monolayers are a = 3.593 Å and 3.549 Å, respectively, and the bond lengths of B-Te, B-B and Pt-S are 2.331, 1.715 and 2.487 Å, respectively, which are in agreement with the peer-reviewed results [34,35].

### 3.1. Electronic Properties of the PtS_2_/BTe vdW Heterostructre

**Figure 2:**
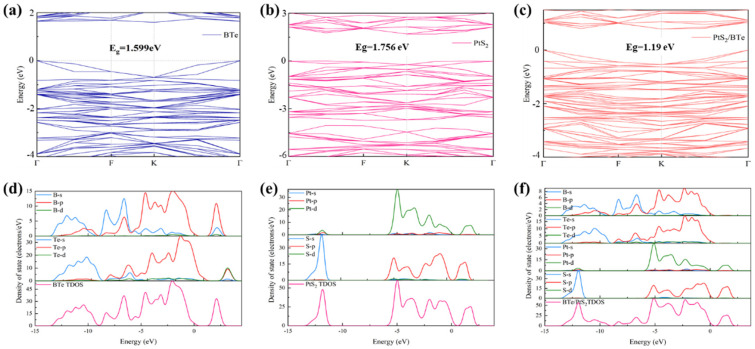
Energy band structures and PDOS of (**a**,**d**) BTe monolayer, (**b**,**e**) PtS_2_ monolayer, (**c**,**f**) PtS_2_/BTe vdW heterstructure.

As depicted in Figure 2, the calculated intrinsic bandgaps for the isolated BTe and PtS_2_ monolayers are 1.599 eV and 1.756 eV, respectively. Specifically, BTe exhibits an indirect bandgap nature (Figure 2a), where the valence band maximum (VBM) and conduction band minimum (CBM) are situated at distinct high-symmetry points in the Brillouin zone (G point for VBM and K point for CBM). Conversely, PtS_2_ acts as a direct bandgap material with both its VBM and CBM aligning at the K point (Figure 2b). Upon assembling the BTe and PtS_2_ supercells into a vdW heterojunction, the composite material maintains a semiconducting profile but with a narrowed bandgap of 1.09 eV. The total and partial densities of states (TDOS and PDOS) for the individual components are plotted in Figure 2d,e. For the BTe layer, the band edges predominantly originate from the B-2p and Te-5p orbitals, whereas the Pt-d orbitals dominate the band edges in PtS_2_. Regarding the heterostructure’s DOS (Figure 2f), the valence region exhibits nine distinct peak clusters: the deep states between −15 eV and −10 eV arise primarily from S-s orbitals, the −10 eV to −5 eV range is governed by B-s orbitals, and the upper valence states (−5 eV to 0 eV) feature hybridized contributions from both layers. Meanwhile, the unoccupied states above the Fermi level are largely dictated by the PtS_2_ monolayer.

### 3.2. Strain and Bandgap

Introducing in-plane biaxial strain is a well-established strategy for tuning the optoelectronic characteristics of nanoscale heterojunctions [36]. To this end, we evaluated the electronic response of the PtS_2_/BTe vdW heterojunction under applied strain, mathematically expressed as

ε = (α − α_0_)/α_0_ = Δα/α_0_ (1)

with α and α_0_ representing the lattice parameters of the strained and pristine configurations, respectively.

**Figure 3:**
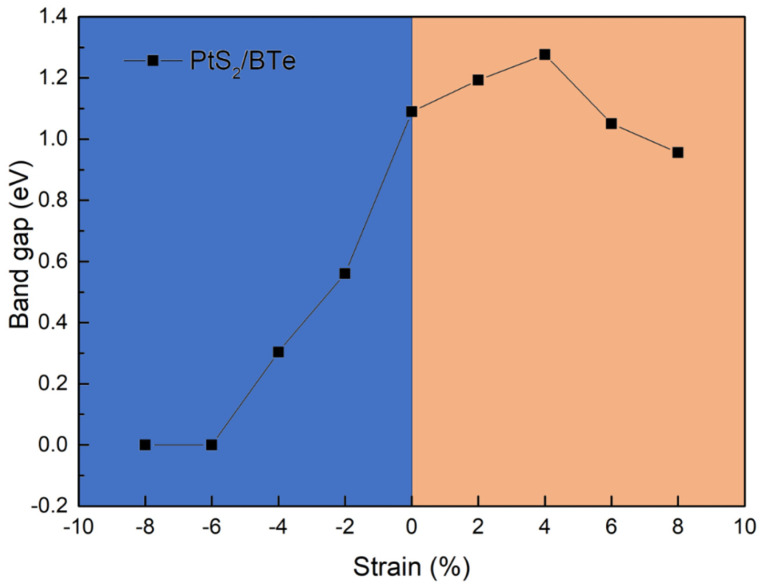
The bandgap under different biaxial strains for PtS_2_/BTe vdW heterojunction.

As illustrated in Figure 3, mechanical deformations significantly alter the bandgap, where negative and positive values correspond to compressive and tensile strains. Under continuous compression, a monotonic reduction in the bandgap is observed. In contrast, under applied tension, the bandgap initially expands before undergoing a subsequent decline. Overall, within the evaluated strain window of −8% to 8%, the bandgap fluctuates between 0 and 1.276 eV. Notably, the most dramatic narrowing of the bandgap dropping from 1.276 eV to 0.956 eV occurs when the tensile strain is intensified from 4% to 8%. The change in bandgap is more obvious, which decreases from 1.276 to 0.956 eV. The bandgap is also more pronounced when the applied tensile stress increases from 4% to 8%, which decreases from 1.276 to 0.956 e V. The bandgap is more pronounced when the applied tensile stress increases from 4% to 8%.

### 3.3. Optical Properties of the Ga_2_SSe/WTe_2_ vdW Heterostructre

**Figure 4:**
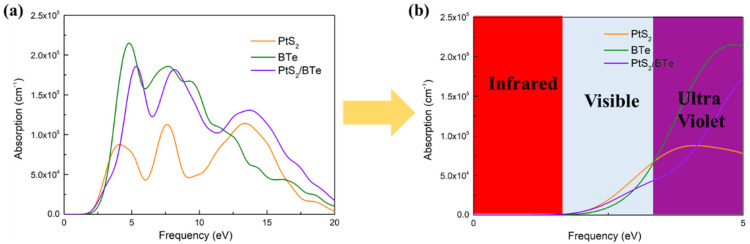
(**a**) Optical absorption of PtS_2_ monolayer, BTe monolayer, along with the PtS_2_/BTe vdW heterojunction. (**b**) The extended absorption spectra in the optical absorption.

The study of light–matter interactions is an important tool for in-depth analysis of the optical properties of semiconductors [37], so the light absorption properties of BTe, PtS_2_ and the PtS_2_/BTe vdW heterojunction were further investigated. Due to the wide bbandgapof BTe, its absorption of visible light is low. The bandgap value of PtS_2_ is smaller than that of BTe, so the light absorption performance of PtS_2_ is stronger than that of BTe. Figure 4a shows that the absorption peaks of PtS_2_ and BTe in the ultraviolet range at wavelengths of 4.03 eV and 4.87 eV are 0.865 × 10^5^ cm^−1^ and 2.441 × 10^5^ cm^−1^, respectively. After the formation of the PtS_2_/BTe vdW heterojunction, the overall optical absorption spectrum is slightly red-shifted, and the absorption intensity in the ultraviolet region of 3.3–5 eV is greatly increased, which can be further enhanced by increasing the light trapping area of the heterojunction or reducing the diffusion distance of the carriers to enhance the absorption of visible light.

**Figure 5:**
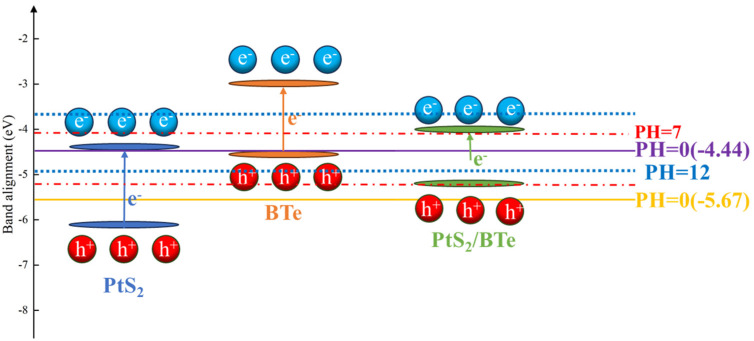
Illustration of the MWH photocatalytic reaction mechanism. In the built-in electric field, blue and red represent the electron aggregation and depletion regions, respectively.

The transfer mechanism of photogenerated carriers in the PtS_2_/BTe vdW heterojunction is shown in Figure 5. When irradiated by visible light, the electrons in the BTe and PtS_2_ layers are photo-excited to jump from VB to CB. The presence of the built-in electric field promotes the compounding of holes in the BTe layer VB and electrons in the PtS_2_ layer CB. Eventually, the electrons in the BTe layer CB and the holes in the PtS_2_ layer VB are left with higher reduction potentials, which significantly increases the redox potentials compared with that of type II heterojunctions, and makes the PtS_2_ layer act as the anode of water electrolysis to undergo oxidation reaction, while the BTe layer acts as the cathode of water electrolysis to undergo reduction reaction. The S-type heterojunction can promote the recombination of weakly reducing electrons and weakly oxidizing holes while spatially separating electrons and holes under strong redox capacity, retaining the strong reducing electrons and oxidizing holes to participate in the reduction and oxidation reactions, respectively [38]. From Figure 5, it can be seen that the redox potentials of PtS_2_ are lower than that of BTe. At pH = 0, 7 and 14, the oxidation potentials of water are −5.56, −5.18 and −4.73 eV, and the reduction potentials of water are −4.44, −4.03 and −3.03 eV, respectively. The oxidation potentials of water are −5.56, −5.18 and −4.73 eV, respectively, and the reduction potentials of water are −4.44, −4.03 and −3.62 eV, respectively. In order to satisfy the requirements of photocatalytic water decomposition, the CBM potential of the photocatalysts should be higher than the standard hydrogen potential, and the VBM potential should be lower than the standard oxygen potential [39]. The CBM potentials of PtS_2_ and BTe are −4.98 and −2.98 eV, and the VBM potentials are −6.10 and −4.59 eV, respectively. When pH = 7, the CBM and PtS_2_ VBM of BTe are at the H^+^/H_2_ and O_2_/H_2_ potentials, respectively, indicating that they can meet the photocatalytic water decomposition requirements at pH = 7 at room temperature. The redox potential of the PtS_2_/BTe vdW heterojunction is enlarged due to the small energy band value in the PBE-GGA general function calculation, which enlarges the positions of the actual oxidation and reduction reactions and meets the requirement of the redox potential for photocatalytic water decomposition, and broadens the range of the photocatalytic water decomposition of pH compared with the two monolayer materials. In conclusion, the PtS_2_/BTe vdW heterojunction can be used as an efficient photocatalytic material due to its excellent photocatalytic water decomposition performance.

Across the ultraviolet-to-visible spectrum, semiconductor materials behave as continuous media, allowing their linear macroscopic optical behavior to be characterized by the complex dielectric function:

(2) $$\varepsilon \left(\omega \right)\;=\varepsilon_{1}\left(\omega \right)+i\varepsilon_{2}(\omega )$$

where ε_1_ and ε_2_ denote the real and imaginary components, respectively.

**Figure 6:**
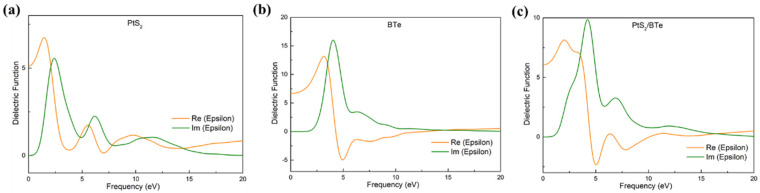
Real part and imaginary part of the dielectric function of (**a**) intrinsic PtS_2_, (**b**) BTe and (**c**) PtS_2_/BTe vdW heterojunction.

These components for the individual monolayers and the assembled vdW heterojunction are plotted in Figure 6. The static dielectric constant, derived from the real part at zero photon energy, reflects the material’s polarization capacity and electron-binding strength under an external electric field. As demonstrated, the static permittivity values for isolated PtS_2_, BTe, and the heterostructure are 5.12, 6.66, and 6.06, respectively. The enhanced value of the heterojunction relative to pristine PtS_2_ implies an augmented ability for charge binding and polarization in the composite system. In the low-energy regime, ε_1_ rises alongside photon energy, peaking at 6.72, 13.2, and 8.1 for the respective systems. Examining the imaginary part (ε_2_), which dictates optical absorption, reveals primary peaks located at 2.46 eV (PtS_2_), 4.65 eV (BTe), and 4.08 eV (PtS_2_/BTe vdW heterojunction). Furthermore, the heterostructure exhibits a progressively stronger absorption response in the visible region as incident photon energy increases, highlighting a significant improvement in the system’s overall light-harvesting capability.

### 3.4. Charge Density Difference and Work Function

**Figure 7:**
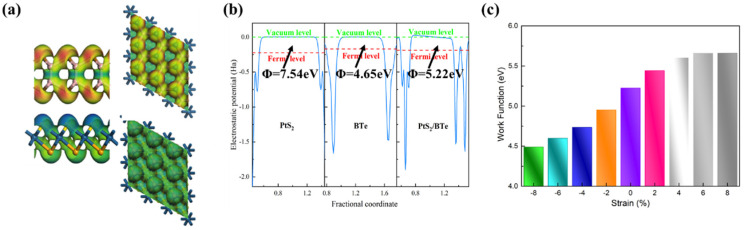
(**a**) The charge density difference in PtS_2_/BTevdW heterostructre. (**b**) The work function of PtS_2_/BTe vdW heterostructre. (**c**) The work function of PtS_2_/BTe vdW heterostructre under strain.

To elucidate the interfacial coupling and charge transfer dynamics within the PtS_2_/BTe vdW heterojunction, we calculated the planar average charge density and electrostatic potential along the out-of-plane (Z) direction. Owing to its inherent structural symmetry, the electrostatic potential of the BTe monolayer displays a highly symmetrical profile. Notably, the potential of the BTe layer sits substantially lower than that of PtS_2_, establishing an interlayer potential difference of 13.06 eV that thermodynamically drives electron migration from the PtS_2_ layer toward the BTe layer. Specifically, the calculated work functions for isolated BTe and PtS_2_ are 4.65 eV and 7.54 eV, respectively. Furthermore, strain engineering assessments (Figure 7c) indicate that the work function scales proportionally with tensile strain, whereas compressive strain induces a continuous reduction in its value. Upon interfacing these two materials, their inherent work function disparity triggers a spontaneous spatial redistribution of charges at the interface. This is visually corroborated by the charge density difference profile in Figure 7a, where the red and blue regions denote charge accumulation and depletion zones, respectively. The accumulation zones are predominantly localized adjacent to the BTe layer, while depletion zones encompass the PtS_2_ side. This distinct charge transfer direction confirms that PtS_2_ acts as the electron donor and BTe acts as the electron acceptor, which is in perfect agreement with our preceding energy band and electrostatic potential analyses.

## 4. Conclusions

In summary, the electronic structure as well as photocatalytic properties of the PtS_2_/BTe vdW heterojunction were systematically investigated in the present work by a first-principles approach. The results show that the PtS_2_/BTe vdW heterojunction has good thermodynamic stability, a bandgap value of 1.19 eV, and is a semiconductor material, showing a staggered band structure, the band edge position across the water redox potential heterojunction interface formed from the PtS_2_ to the BTe built-in electric field, which is conducive to the effective separation of electron–hole pairs. The photo-absorption coefficient of the PtS_2_/BTe vdW heterojunction in the visible region reaches a 105 cm^−1^ order of magnitude, and the carriers still have a strong redox ability under the biaxial strain from −8% to 8%, so the PtS_2_/BTe vdW heterojunction is a good semiconductor material for photocatalysis materials.

## Figures and Tables

**Table 1:** The bond length and bandgap of monolayer BTe and PtS_2_ monolayer.

	Bond Length/Å		Bandgap/eV	
	Present Work	Previous Work	Present Work	Previous Work
BTe	2.331	2.472 ^a^	1.599	1.53 ^a^
PtS_2_	2.487	2.487 ^b^	1.756	1.775 ^b^

^a^ Ref. [34]. ^b^ Ref. [35].

## Data Availability

The original contributions presented in this study are included in the article. Further inquiries can be directed to the corresponding author.
